# Decision tree-based method for integrating gene expression, demographic, and clinical data to determine disease endotypes

**DOI:** 10.1186/1752-0509-7-119

**Published:** 2013-11-04

**Authors:** ClarLynda R Williams-DeVane, David M Reif, Elaine Cohen Hubal, Pierre R Bushel, Edward E Hudgens, Jane E Gallagher, Stephen W Edwards

**Affiliations:** 1 National Health and Environmental Effects Research Laboratory – Integrated Systems Toxicology Division, U.S. Environmental Protection Agency, Research Triangle Park, Durham, NC 27711, USA; 2 National Center for Computational Toxicology, U.S. Environmental Protection Agency, Research Triangle Park, Durham, NC 27711, USA; 3 Biostatistics Branch, National Institute of Environmental Health Sciences, Research Triangle Park, Durham, North Carolina 27709, USA; 4 National Health and Environmental Effects Research Laboratory – Environmental Public Health Division, U.S. Environmental Protection Agency, Research Triangle Park, Durham, NC 27711, USA; 5 Present address: Julius L. Chambers Biomedical/Biotechnology Research Institute, Biology Department-North Carolina Central University, Durham, NC 27707, USA; 6 Present address: Biological Sciences Department, North Carolina State University, Raleigh, NC 27695, USA

**Keywords:** Asthma, Endotypes, Gene Expression, Integrated analysis

## Abstract

**Background:**

Complex diseases are often difficult to diagnose, treat and study due to the multi-factorial nature of the underlying etiology. Large data sets are now widely available that can be used to define novel, mechanistically distinct disease subtypes (endotypes) in a completely data-driven manner. However, significant challenges exist with regard to how to segregate individuals into suitable subtypes of the disease and understand the distinct biological mechanisms of each when the goal is to maximize the discovery potential of these data sets.

**Results:**

A multi-step decision tree-based method is described for defining endotypes based on gene expression, clinical covariates, and disease indicators using childhood asthma as a case study. We attempted to use alternative approaches such as the Student’s t-test, single data domain clustering and the Modk-prototypes algorithm, which incorporates multiple data domains into a single analysis and none performed as well as the novel multi-step decision tree method. This new method gave the best segregation of asthmatics and non-asthmatics, and it provides easy access to all genes and clinical covariates that distinguish the groups.

**Conclusions:**

The multi-step decision tree method described here will lead to better understanding of complex disease in general by allowing purely data-driven disease endotypes to facilitate the discovery of new mechanisms underlying these diseases. This application should be considered a complement to ongoing efforts to better define and diagnose known endotypes. When coupled with existing methods developed to determine the genetics of gene expression, these methods provide a mechanism for linking genetics and exposomics data and thereby accounting for both major determinants of disease.

## Background

Complex diseases, caused by multiple genetic and environmental factors acting together, are often difficult to diagnose, treat, and study due to the limited understanding of their mechanistic underpinnings. New approaches are needed to better understand the biologic processes that underlie the clinical phenotypic expression of complex disease [1]. Study of the associations between bioindicators and disease outcomes can provide evidence of early changes preceding disease, which leads to a better understanding of the mechanistic underpinnings of complex disease [2].

Gene expression microarrays developed over the past decade represent powerful tools for investigating biological and disease processes [3]. For example, Sen [4] summarized the published literature on transcriptional profiles resulting from exposure of cells or organisms to complex environmental mixtures such as cigarette smoke, diesel emissions, urban air, motorcycle exhaust, carbon black, jet fuel, and metal ore fumes. Relevant to this study are studies using blood cell gene expression analyses to evaluate environmental chemical exposures [5-7]. Lobenhofer et al. [8] demonstrated the utility of performing gene expression profiling on whole blood samples as an effective surrogate for the target organ to classify compound exposures. They demonstrated the power to detect not only the presence of liver injury, but also the potential severity. A significant body of literature now provides the promise of gene expression in peripheral blood cells for developing biomarkers of disease [9-11]. Together, these studies suggest that gene expression in peripheral blood cells has a role in deciphering target tissue effects relevant to both exposure and clinical assessments. Gene expression studies with “phenotypic anchoring” are limited but provide more meaningful results than when either gene expression or phenotypic data is viewed alone [8,12-14]. Few studies of childhood asthma use gene expression in peripheral blood cells; however, such data combined with clinical measurements may provide a more comprehensive understanding of asthma etiology and diagnosis.

Childhood asthma is known to involve both genetic and environmental components with low-level, ubiquitous environmental exposures contributing substantially [15,16]. Evidence exists for genomic features contributing to pathogenesis, which in part relates to innate immunological characteristics such as variation in host defense genes [17]. Some scientists believe that, as the result of rapid urbanization over the last few decades, genes previously protecting humans from parasitic infection may now contribute to a 'misdirected’ response to environmental agents [18]. Tools to characterize and unravel interacting genetic and environmental factors are clearly required [19,20]. Understanding the contribution of environmental factors to disease susceptibility requires a more comprehensive view of exposure and biological response than has traditionally been applied.

Various methods have been proposed to identify and describe the mechanistic underpinning of asthma and complex airway disease endotypes. Several groups have defined asthma endotypes based on phenotypes and putative pathophysiology that was applied to asthma for use in clinical study design and drug development [21-25]. Cho et al. used clustering algorithms to better define airway disease, without specifically addressing asthma, by combining genetic and phenotypic variables with a combination of factor and cluster analysis [26]. However, with childhood asthma in particular, the phenotypes are variable, leading to difficulty in linking phenotype information to endotypes of asthma [27].

Clustering was most successful when applied to severe asthmatics [28,29]. Weatherall et al. defined distinct clinical phenotypes of airway disease (including asthma) by cluster analysis [30]. Kelley et al. suggested grouping patients based on questionnaire responses and skin prick tests followed by multivariate regression to determine whether demographic or potential risk factors varied between phenotypes and whether measures of severity varied by phenotypes [31]. Kelley’s method was successful in defining endotypes but did not lead to greater mechanistic understanding. Results from the National Heart, Lung, and Blood Institute’s Severe Asthma Research Program have shown clustering to be very effective generally [29] and specifically for children [28]. These methods rely heavily on existing clinical criteria and are clearly the best approach when determining diagnostic criteria for asthma endotypes. However, restricting focus to established clinical criteria is not a viable option when the primary goal is the discovery of potentially novel mechanisms.

Auffray et al. proposed the use of global genome, transcriptome, proteome, and metabolome data sets collected in cross-sectional patient cohorts and integrated with biologic and clinical data to develop predictive multi-scale models, which can be evaluated and expanded using systematic perturbations of carefully selected animal models of disease [1]. Many recent studies illustrate the feasibility of this approach [32]. Gene expression from airway epithelial cells was used to define potential asthma endotypes [33], which had implications for corticosteroid treatment [34] and biomarker identification [35]. Similar studies using induced sputum samples [36], and blood [37,38] have also provided information about molecular mechanisms underlying different asthma endotypes.

Current methods have been most successful when characterizing disease using previously established biomarkers or looking at gene expression in the target tissue such as airway epithelial cells. In this paper, we present a new approach focused specifically on the identification of novel biomarkers and previously unreported mechanisms driving disease endotypes from blood. This method was developed using data from the Mechanistic Indicators of Childhood Asthma (MICA) study [39]. The new method was developed as a result of the lack of success with traditional methods such as Student’s t-test, single-data domain clustering, and the more complex Modk-prototypes algorithm. We evaluate the results from each of the analysis methods based on segregation of known asthmatics from non-asthmatics, as well as information provided to support biological interpretation of the results. Secondly, we evaluate the impact on the analytical results of how and when each domain of data is incorporated in each analysis scheme. Insights of analysis methods with respect to complex disease are presented.

## Methods

### Study design

This study involved data collected from participants of the Mechanistic Indicators of Asthma (MICA) study, conducted by the United States Environmental Protection Agency between November 2006 and January 2007 in Detroit, Michigan, as described [39]. This was a cross-sectional study based on a stratified sample of children with asthma and children without asthma, selected in an approximately 1:1 ratio. A total of 205 children age 9–13 years old were enrolled. The study design and protocols were approved by the Institutional Review Boards at Henry Ford Health System (Detroit, MI), Westat Inc. (Rockville, MD), and the University of North Carolina at Chapel Hill (Chapel Hill, NC – US EPA’s IRB of record). Written consent was obtained from guardians, and written assent was obtained from each child, with an oral review of both consent and assent prior to study enrollment. According to self-reported race, study subjects were 85% African American [39]. Data were collected from subjects via clinical measurements; observational evaluations by clinicians; and questionnaires distributed to the parents of subjects. Data pertaining to subjects’ demographic, physical activity, and medical history were collected through a questionnaire distributed to the parents of subjects. Gene expression data were compiled to evaluate potential biomarkers of asthma.

### Clinical and demographic covariates

Within this study, “covariate” (Table 1) describes every type of data collected except for gene expression and indicators of health outcome, with the latter being asthma and allergy status. Clinical measurements collected from blood and lung function test are referred to as clinical covariates, whilst data collected via the questionnaire and clinician observations, except for indicators of health outcome, are referred to as demographic covariates. Covariates consist of a number of clinical measures of hematologic, immunologic and cardiopulmonary variables, body size measures, indicators of allergy and asthma, and concentration and/or percent distribution of white blood cells (i.e. eosinophils, monocytes, lymphocytes, neutrophils (Table 1). During the clinic visit, clinicians evaluated subjects and collected biospecimens for clinical tests [39].

**Table 1:** List of 81 covariates used in analysis

**Category**	**Description**	**Units**
Allergen screen	**FoodScreen (5 food allergens)	kUA/L
	**Phadiatop (15 aeoallegens)	kUA/L
	Total Serum IgE	kU/L
Blood Chemistry	C-reactive Protein	mg/ml
	Albumin	g/dL
	Alkaline Phosphatase	IU/L
	Serum Glutamic Pyruvic Transaminase	IU/L
	Serum Aspartate Aminotranferase (AST) Serum Glutamic-Oxaloacetic Transaminase (SGOT)	IU/L
	Albumin/Globulin ratio	
	Serum Total Bilirubin	mg/dL
	Serum Blood Urea Nitrogen	mg/dL
	Serum Blood Urea Nitrogen Creatinine Ratio	
	Serum Calcium	mg/dL
	Serum Chloride	mmol/L
	Serum Creatinine	mg/dL
	Serum Ferritin	ng/ml
	Serum Fibrinogen	mg/dL
	Serum Gamma-Glutamyl Transpeptidase (GGT)	IU/L
	Serum Total Globulin	g/dL
	Blood Hematocrit	%
	Blood Hemoglobin	g/DdL
	Serum Iron	ug/dl
	Serum Lactate Dehydrogenase	IU/L
	Plasma Leptin	ng/ml
	Serum Glycated Hemoglobin	%
	Serum Glucose	mg/dL
	Potassium	mmol/L
	Urine Creatinine	mg/dl
	Serum Arachidonic Acid	ug/ml
	Serum Osmolality	mOsmol/kg
	Serum Phospholipids Concentration	Mg/dL
	Serum Phosphorus	mg/dL
	Serum Total Protein	g/dL
	Sodium	mmol/L
	BP Oxygen Saturation (Dissolved Oxygen)	%
CBC	White Blood Cell Count	K/uL
	Basophil percent of sum White Blood Cells	%
	Eosinophil percent of sum White Blood Cells	%
	Lymphocyte percent of sum White Blood Cells	%
	Monocyte percent of sum White Blood Cells	%
	Neutrophils percent of sum White Blood Cells	%
Clinic	Subject Age	Years
	Subject Height	Cm
	Subject Body Mass Index Weight/height	kg/m^2^
	Subject Weight	Kg
	Mean of first Two Diastolic Blood Pressure Measurements	mmHg
	Mean of first two Systolic Blood Pressure Measurements	mmHg
	Blood Pressure Pulse	beats/min
Hematology	Red Blood Cell Count	M/uL
	Platelet Count	K/uL
	Mean Corpuscular Hemoglobin	Pg
	Mean Corpuscular hemoglobin concentration	g/dL
	Mean Corpuscular Volume	fL***
	Red Blood Cell Distribution Width	%
Inflammatory	Interleukin-4	pg/ml
	Serum Total Antioxidant Status	mmol/L
	Serum Unbound Iron-Binding Capacity	ug/dl
	Serum Uric Acid	mg/dL
	Plasma Average of Reactive Oxygen Species Measurements minus Control	RLU****
Lipids	High density Lipoprotein	mg/dL
	Low density Lipoprotein	mg/dL
	Total Cholesterol to High density Lipoprotein Ratio	
	Total Cholesterol	mg/dL
	Serum Triglycerides	mg/dL
	Very Low Density Lipoprotein	mg/dL.
Lung Function	Forced Expiratory Flow Between 25% and 75% of Forced Expiratory Flow	
	Fractional Exhaled Nitric Oxide	ppb
	Forced Expiratory Volume /ratio to Forced Vital Capacity	%
	Peak Expiratory Flow	(L/min)
Serum Allergens	*Serum Alternaria Alternata	kUA/L
	*Serum Aspergillus Fumigatus	kUA/L
	*Serum Cat Dander Epithel	kUA/L
	*Serum Cladosporium Herbarum	kUA/L
	*Serum Derm Farin Dustmite	kUA/L
	*Serum Derm Pter Dustmite	kUA/L
	*Serum Dog Dander	kUA/L
	*Serum German Cockroach	kUA/L
	*Serum Mouse Urine Protein	kUA/L
	*Serum Penicillium Notatum	kUA/L
	*Serum Rat Urine Protein	kUA/L

*Indicates variables not included in the 67 Covariate List. **Indicates variables that were converted to categorical variables for the 67 Covariate List.***femtoliters. ****relative luminescence unit.

Each covariate incorporated into the analysis was selected by a panel of multidisciplinary experts from statistics, systems biology, epidemiology and toxicology based solely on the data characteristics. Given the focus on discovery, no a priori clinical criteria were applied. The inclusion criteria were completeness (lack of missing data), sampling distribution (normality was checked before correlations were calculated), and comparability with accepted data values based on established clinical criteria (where available) or data from previous studies. Based on these criteria, a set of 81 continuous covariates was selected for consideration in each of the data analysis methods, including blood chemistry of clinical indicators of allergic disease (Table 1). Care was taken to remove values with a common derivation (such as a value calculated from a different covariate), but some correlated biomarkers were retained to fully represent the potential biology of the system.

**Figure 1:**
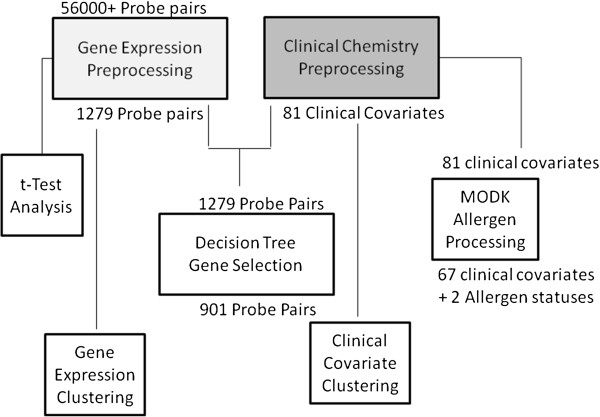
Data pre-processing workflow for gene expression and clinical data used in each of the analysis methods.

The 81 covariates were used for single-domain clustering and multi-step decision tree methods. Further processing of the clinical covariates was performed to remove indicators of allergic disease for the second clinical covariate single-domain clustering and Modk-prototype algorithm methods as described below (Figure 1).

As discussed previously, childhood asthma is highly variable in presentation; therefore two indicators of asthma disease outcome were considered. That is, a child was considered asthmatic if (1) the clinical records showed one or more asthma-related emergency department visits, two or more asthma-related outpatient visits, or two or more asthma-related medications and (2) the child’s parent reported a physician’s diagnosis from the question “Has a doctor ever diagnosed your child with asthma” included on the MICA questionnaire. Subjects with conflicting or incomplete asthma status data were excluded from the analysis, resulting in 146 subjects with known asthma status. Subjects reported to have had an asthma episode within the last 12 months were also considered when the methods were able to accept multiple input indicators of asthma.

### Gene expression

Blood collected during observational clinic visits was used for gene expression analysis [39]. Gene expression was measured using the Affymetrix GeneChip® Human Genome U133 Plus 2.0 Array by Expression Analysis, Inc. (http://www.expressionanalysis.com; Durham, NC). Before target production, the quality and quantity of each RNA sample was assessed using a 2100 BioAnalyzer (Agilent). Target was prepared and hybridized according to the "Affymetrix Technical Manual". Total RNA (5 ug) was hybridized with four PNA oligomers whose sequences are complementary to the 3’ portions of the alpha and beta hemoglobin RNA transcripts. The PNA oligomers form stable duplex structures with the globin mRNA and block the progression of reverse transcriptase. The inhibition of globin cDNA synthesis dramatically reduces the relative amount of anti-sense, biotin-labeled cRNA corresponding to the hemoglobin transcripts (http://www.expressionanalysis.com/images/uploads/tech_notes/Globin_Tech_Note_final_v2.pdf). RNA was converted into cDNA using Reverse Transcriptase (Invitrogen) and a modified oligo (dT)24 primer that contains T7 promoter sequences (GenSet). After first strand synthesis, residual RNA was degraded by the addition of RNaseH and a double-stranded cDNA molecule was generated using DNA Polymerase I and DNA Ligase. The cDNA was then purified and concentrated using a phenol:chloroform extraction followed by ethanol precipitation. The cDNA products were incubated with T7 RNA Polymerase and biotinylated ribonucleotides using an In Vitro Transcription kit (Affymetrix). The resultant cRNA product was purified using an RNeasy column (Qiagen) and quantified with a spectrophotometer. The cRNA target (20 ug) was incubated at 94°C for 35 minutes in fragmentation buffer (Tris, MgOAc, KOAc). The fragmented cRNA was diluted in hybridization buffer (MES, NaCl, EDTA, Tween 20, Herring Sperm DNA, Acetylated BSA) containing biotin-labeled OligoB2 and Eukaryotic Hybridization Controls (Affymetrix). The hybridization cocktail was denatured at 99°C for 5 minutes, incubated at 45°C for 5 minutes and then injected into a GeneChip cartridge. The GeneChip array was incubated at 42°C for at least 16 hours in a rotating oven at 60 rpm. GeneChips were washed with a series of non-stringent (25°C) and stringent (50°C) solutions containing variable amounts of MES, Tween20 and SSPE. The microarrays were then stained with Streptavidin Phycoerythrin and the fluorescent signal was amplified using a biotinylated antibody solution. Fluorescent images were detected in a GeneChip® Scanner 3000 and expression data was extracted using the GeneChip Operating System v 1.1 (Affymetrix). All GeneChips were scaled to a median intensity setting of 500.

The raw microarray data were subjected to the Reduction of Invariant Probes (REDI) algorithm to remove data from unresponsive probes (http://www.expressionanalysis.com/images/uploads/tech_notes/REDI_Tech_note_final_v2.pdf). The data were then normalized using the MAS 5.0 software [40]. Principal Components Analysis (PCA) identified a significant sex effect on gene expression globally, which might dominate the gene expression-covariate correlations, so a standard linear adjustment for the effect of sex was performed, where the residuals were used for subsequent analysis. Additional justification for controlling the effect of sex arises out of the age range of our subjects (9–12 years of age), which spans the national average age at menarche. Next, genes with lower variation across our subjects were filtered out of the gender-adjusted data by selecting only probe sets with an inter-quartile intensity range (IQR) greater than 2000. This IQR level was identified as the inflection point in the empirical cumulative distribution of 54675 probe sets having a significant departure from baseline (See Additional file 1: Figure S1). Exploring a broad set of IQR thresholds showed that the chosen value of 2000 allowed diverse genes to pass the filter (i.e. more than just housekeeping genes of extremely high expression) while avoiding genes with trace expressional levels that are prone to spurious ratio-based associations at later steps. Finally, the microarray data were log2 transformed to pull data into a roughly Gaussian distribution. Of the more than 56000 probe sets collected for each subject, a subset of 1279 were selected for use in each of the methods as described below. Further steps were necessary for the gene expression input of the multiple-step decision tree method (Figure 1).

The microarray data from this publication have been submitted to the Gene Expression Omnibus (GEO) repository (http://www.ncbi.nlm.nih.gov/geo/) and assigned the identifier GSE35571.

### Analysis methods

**Figure 2:**
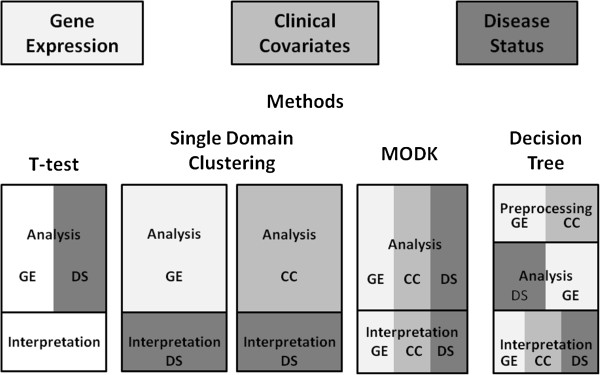
**Data incorporation scheme for each of data analysis method consideration.** Shading corresponds to each data domain: Gene Expression, Clinical Covariates, and Indicators of Disease Status.

Each data analysis method involved different domains of data at different steps from preprocessing and analysis to interpretation (Figure 2) as described below.

#### Traditional methods

Each domain of data was evaluated using 3D scatterplots and PCA to determine if there were any outliers. Two observations were noted 1) simple dimension reduction of repetitive or redundant measures was not possible and 2) one subject appeared to be an outlier in the covariate data but not in the gene expression data. As such no outliers were removed. A two-sample t-test procedure was performed using R multtest package to determine the consistency of asthma diagnosis using the null hypothesis that there was no significant difference between the gene expression intensities of asthmatics and non-asthmatics with the dataset (http://cran.r-project.org/web/packages/multtest/index.html). The t-test procedure was performed using each asthma definition yielding two lists of differentially expressed genes at α=0.05. Results were evaluated without multiple testing correction.

As single domain clustering has previously been shown as a successful method for understanding disease etiology, single domain clustering was performed. The ClusterSim R package was used to search for the optimal clustering procedure for each domain of data. ClusterSim identifies the combination of normalization method, distance metric and classification method that produces the highest cluster validity index score considering 2 to 50 possible clusters (http://cran.r-project.org/web/packages/clusterSim/clusterSim.pdf). The ClusterSim R package considers the “kmeans”, "single", "complete", "average", "mcquitty", "median", "centroid", "ward", "pam", and "diana" classification methods. For these data, the algorithm considers the manhattan, euclidean, chebychev (max), squared euclidean, generalized distance measure 1, Canberra, and Bray-Curtis distance measures. The top three clustering combinations correspond to the best values of three internal cluster validity index scores: maximum Rousseeuw’s Silhouette cluster quality index [41], maximum Baker and Hubert adaptation of Goodman and Kruskal’s Gamma statistic [42], and minimum Hubert and Levine’s internal cluster quality index [43]. The clustering was a result of maximizing the cluster validity index values and not as the result of outliers.

Given a sample size of 146 subjects, clustering combinations that produce large numbers of clusters, e.g., 50, offer limited ability to learn mechanistic information because each cluster is limited to a small number of subjects, e.g., three. Conversely, clustering combinations that produce a small number of clusters, e.g., two, are difficult to interpret because the variability of each gene or covariate is higher within clusters having a large number of subjects, e.g., 75, presumably because different endotypes have been combined in the cluster. Resulting plots of clusters were examined visually for separation of asthmatics and non-asthmatics as well as cluster separation

A similar process was used for clinical and allergic disease indicator covariates considering 2 to 25 possible clusters. Two sets of covariates were used, 81 covariates and a subset of 67 covariates. The subset of 67 covariates was considered for comparison to the Modk-prototype algorithms method (refer to Methods: Modk for details). The top three methods were used to determine the best clustering, and the covariate data were processed as such using the identified methods in R. Resulting plots of clusters were examined visually to evaluate the different clustering methods with respect to the separation of asthmatics from non-asthmatics.

#### Modk

The Modk-prototypes data analysis strategy was a multi-step process that considered the gene expression, clinical, and disease indicator covariates simultaneously in a meaningful disease context [12,44]. The original implementation of the Modk-prototypes algorithm partitions subjects into their respective disease class groups using k-means and k-modes clustering over three subjectively weighted data domains: target organ gene expression, clinical chemistry, and histopathology. This approach was adapted for our study by replacing target organ gene expression with blood gene expression and using allergy and asthma health outcome indicators in place of histopathology calls.

The first step of the Modk-Prototypes data analysis strategy was to separate the clinical covariates from the indicators of allergic health outcome (Table 2). Of the 81 covariates (Table 1), 67 remained as continuous covariates. The remaining 14 covariates were summarized into two indicators of allergic disease: phadiatop and foodscreen. Phadiatop summarizes the subject’s propensity to exhibit allergic disease due to environmental allergens and foodscreen summarizes the subject’s propensity to exhibit allergic disease due to food allergens. Phadiatop and foodscreen were transformed into categorical variables with five levels with the highest level being highly allergic and the lowest level being not allergic. If a subject was positive for any of the serum allergens (Table 1), a positive test for atopy from the panel of allergens (Phadiatop test) was verified to insure that no information was lost. The allergy levels were based on the quantiles of the two measurements (See Additional file 1: Table S1).

**Table 2:** Indicators of disease status

**Indicator of Disease**	**Description**	**Known/Unknown**
Confirmed asthma	When parent questionnaire response to the question “has a doctor ever diagnosed this child as having asthma” was confirmed through administrative records regarding clinic visits and the prescription of asthma medication	146/59
Current asthma	Labeled Asthmatics and Non-Asthmatics reporting an asthma attack in the last 12 months	192/13
Questionnaire-defined asthma	Positive response to “has a doctor ever diagnosed this child as having asthma” on parent questionnaire	186/19
Phadiatop – Atopy/Allergen screen	Positive serum test to a panel of at least 15 common allergens	173/32
Foodscreen – Food allergen screen	Positive serum test to a panel of 6 common allergy provoking foods (cows milk protein, egg white wheat codfish peanut and soybean)	190/15

Column 2 provides a description of each disease indicator. Column 3 shows the number of subjects for which a given disease status was unknown.

The nature of the Modk-prototypes algorithm required the serum allergens to be summarized as categorical variables whereas the decision tree method did not. More specifically, many of the serum allergens had a high percentage of imputed values where the raw measurements were below a detectable threshold. Due to the imputation method used, this resulted in the same value for all individuals where the actual value was below the detection limit, which could artificially inflate the measured associations among those variables. In addition, most of the allergen measurements were very highly correlated (i.e. a subject that has a significant value for one allergen tends to have a significant value for another allergen throughout the dataset), which could overweight the influence of the allergen measurements on the final clustering. As described previously, the decision tree method used the full 81-covariate set rather than the 67-covariate subset used in the Modk method. In that case, the gene/covariate associations were calculated separately from the classification of the asthmatics, which reduced the influence of the serum allergens by collapsing them into a single gene cluster.

The next step was to impute all missing continuous values. There were no missing values in the gene expression data. Missing clinical values due to missing blood samples were imputed using mean imputation in the e1071 R package (http://cran.r-project.org/web/packages/e1071/index.html). There were 186 total missing values for 47 out of 67 variables, 2% missingness. There were no consistent missing values across the subjects. For the 47 variables with missing values, there was on average 4 missing values. Upon evaluation, imputed values did not contribute to clustering results and were only necessary to meet the requirements of the Modk-prototypes algorithm. Next, the final dataset was combined in a tabular format with the three indicators of asthma health outcome and two indicators of allergy health outcome (Table 2) followed by 67 clinical covariates, and 1279 gene expression values for each subject annotated to denote the data domain of each variable.

One of the advantages of using this Modk-prototypes algorithm is that the data domains can be weighted adaptively or subjectively. Subjective weighting was based on the 15 “expert suggested weighting schemes” from Bushel et al. [12]. Weighting schemes where the gene expression or clinical chemistry were 0% were removed as well as weighting schemes that were within less than 10% of one another. In all, results shown are for seven separate summative weighting schemes including an adaptive weighting scheme where the weight of each data domain is determined within the algorithm and adjusted at each iteration of the algorithm to maximize the objective function based on cluster quality (Table 3) [44]. Specifically, the objective function is constructed with the sum of the squared Euclidean distances for numeric microarray and clinical chemistry data and simple matching for categorical values in order to measure dissimilarity of the samples. The Modk-algorithm was implemented in Matlab with slight modifications from the original code to allow larger datasets to be processed across multiple workstations in a parallel manner (available upon request from PRB). One run of the Modk algorithm at a specified weighting scheme produced a gene expression, clinical covariate, and indicator of disease prototype for each cluster. The segregation accuracy of each weighting scheme was calculated together with the number of predicted classes of asthmatics and non-asthmatics.

**Table 3:** Modk-prototypes weighting schemes

**Gene expression**	**Clinical covariates**	**Indicators of disease status**
33	33	33
20	40	40
40	20	40
50	50	0
30	60	10
60	30	10
40	40	20
Adaptive	Adaptive	Adaptive

The Modk algorithm was run with 7 pre-defined weighting schemes. This table shows the combinations of gene expression, clinical covariates, and disease indicator weighting for each run. For the adaptive weighting scheme, the weights were determined by the algorithm as described in the methods.

Next, the best-suited weighting schemes were selected based on the segregation accuracy. The prototypes for gene expression and clinical covariates were standardized to z-scores. Subsequently, statistically significant biomarkers, defined by α=0.05, and significant clinical biomarkers, defined as clinical covariates that fall more than 1 standard deviation from their overall mean, were identified. Gene covariates were considered in the same manner.

#### Decision tree

**Figure 3:**
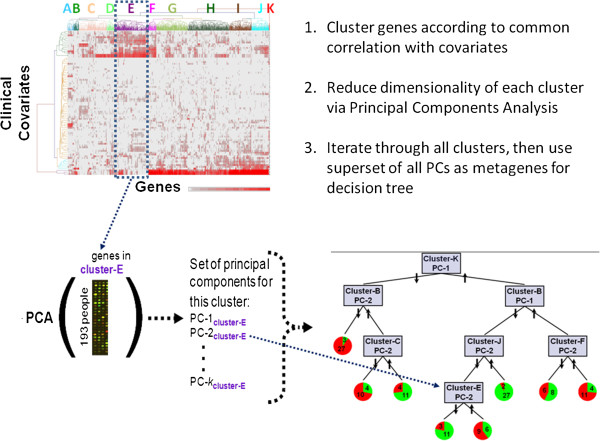
Multistep decision tree method.

The multi -step decision tree method was implemented in R and consists of four sequential steps (Figure 3). The first step was to calculate the correlation of each covariate with each gene expression variable, which resulted in a distribution of Pearson correlation test statistics (81 covariates × 1279 IQR-filtered genes). In order to correct for the multiple covariate tests, we applied a p-value threshold that included a Bonferroni correction of 0.05/81=0.0006, which roughly corresponds to a correlation greater than 0.23. This process resulted in a list of 901 genes whose expression was significantly associated with at least one of the 81 covariates selected for this analysis.

Second, to reveal patterns in the gene-covariate correlation, unsupervised hierarchical clustering (complete linkage) was performed using the absolute correlation values [0, 1] for 901 significantly covariate-associated genes. Clustering revealed eleven groups of genes in which mRNA expression is related to distinct sets of covariates.

Third, the genes in these eleven groups/clusters were used as the basis for constructing metagenes that summarize information content across all gene expression variables in each of the cluster groups. The metagenes were built by performing eleven separate principal components analyses (PCA) on the expression data for each gene cluster identified above. All principal components (PCs) explaining at least 5% of the variance for a cluster were considered in subsequent steps. Each of these PCs was then considered a metagene (because it summarized the expression of a set of genes) in all subsequent analyses. Formally, each metagene is a vector that summarizes the combination of weights (loadings) by which the gene expression variables in that cluster are multiplied to yield subject-wise PCs. For the eleven clusters in Supplemental Figure 1 (See Additional file 1), the number of significant metagenes (PCs meeting the minimum variance criterion) for clusters A through K, respectively, were: 3, 6, 4, 2, 3, 3, 3, 4, 2, 2, 2. Metagenes were named according to gene-covariate cluster and principal component number (e.g. F-2 for the second principal component (PC) from the PCA on genes in cluster F). While the first PC explains the most variance in gene expression, lower PCs (exceeding our variance threshold) were retained in order to capture different aspects of cluster variance.

Finally, in order to identify asthma endotypes, decision trees were built from the meta-genes to segregate subjects according to covariate-associated gene expression patterns. The Weka implementation of the C4.5 algorithm was used (http://www.cs.waikato.ac.nz/ml/weka/) [41,45]. Trees were built to segregate subjects based on asthma status, and a minimum of 14 subjects per terminal leaf was specified (chosen to assure that leaves contained at least 10% of the total subject pool). This recursive partitioning algorithm proceeds by identifying the attribute (metagene) whose split provides the greatest Information Gain (reduction in entropy of asthma status) across all subjects. Starting with the initial split, the algorithm continues growing a tree whose branches are defined by metagenes until splits no longer improve Information Gain or result in leaves containing fewer than 14 subjects. An estimate of predictive ability was obtained by running 10,000 bootstrapped (resampled with replacement) samples through the tree. While alternative tree construction methods, such as Random Forests [46], could have been used, standard decision trees were chosen to keep the focus on our goal of interpretability. Alternative methods that rely on bagging or boosting generate aggregate models that must be statistically parsed to obtain final models, while the trees built here provide a straightforward model of meta-gene interactions.

## Results and discussion

We have developed a new method to address situations where discovery of novel biological mechanisms is the primary focus. Approaches such as the one described here should complement those used to build better diagnostic criteria based on known clinical criteria as our results should provide novel biomarkers for that purpose. With this discovery focus in mind, our new multi-step decision tree was evaluated as an alternative to traditional methods.

Three domains of data were considered: 1) gene expression; 2) clinical covariates; and 3) indicators of health outcomes.

### Traditional methods

A two-sample t-test procedure was performed for each of two asthma indicator variables (Table 2 - Current Asthma, Confirmed Asthma) to test the null hypothesis of no significant difference between the gene expression intensities of asthmatics and non-asthmatics for the 146 subjects for 1279 genes selected for analysis. After correcting for multiple testing, no genes met the significance threshold for either case. Comparing the uncorrected gene lists showed very little overlap between the two lists. Results with the T test might have been improved by accounting for differences in the underlying cell types across samples. However, it has previously been established that asthma is a syndrome consisting of multiple, mechanistically distinct endotypes [23,25,47,48]. Without previous knowledge of how the subjects group by endotype, it is likely that variability associated with mechanistic differences among subjects would be at least as great as that due to changes in cell type. In addition, since the changes in cell type might themselves be indicators of mechanistic differences among the asthmatics, accounting for these differences during the interpretation phase was deemed more appropriate.

Previously, clustering has been successful in defining subtypes of complex disease [22,26,28,29,33,36]. Often the limitation of clustering is the inability to choose the best parameters and clustering methods. For this reason, the combination of normalization method, distance metric, and classification method was selected to produce the highest cluster validity index score. The three most optimal combinations were determined using three internal cluster quality index values: 1) maximum Rousseeuw’s Silhouette cluster quality index [41]; 2) maximum Baker and Hubert adaptation of Goodman and Kruskal’s Gamma statistic [42] and 3) minimum Hubert and Levine’s internal cluster quality index [43]. Additional combinations were evaluated that produced more reasonable numbers based on *a priori* information for determining endotypes of childhood asthma. Single-domain clustering was performed separately for each continuous domain, gene expression and clinical covariate.

**Figure 4:**
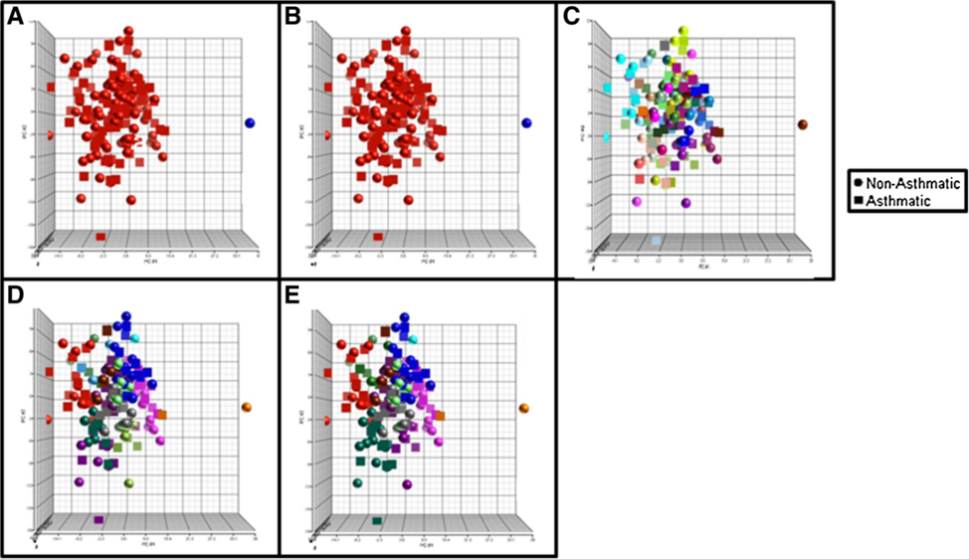
**Scatterplots for each of the Gene Expression Clustering methods (Table**2**); A: Silhouette, B: Baker & Hubert, C: Hubert & Levine, D-E: Additional clustering Combinations.** Colors are representative of individual clusters.

Cluster validity testing suggested that the best gene expression clustering occurred with 2 or 50 clusters depending on the distance measure and clustering method used (Table 4). As shown in Figure 4A-B, the Silhouette and Baker &Hubert clusterings resulted in two groups, one composed of all subjects except one with no separation between asthmatics and non-asthmatics. The Hubert & Levine clustering resulted in 50 non-distinct groups composed of both asthmatics and non-asthmatics (Figure 4C). Further exploration of clustering combinations (summarized in Table 4) yielded similar results (Figure 4D-F). The clusters were not distinct for the number of asthmatics and non-asthmatics or levels of allergic disease (not shown).

**Table 4:** Top ranking gene expression clustering methods from clusterSim

**Index metric**	**Index value**	**Distance measure**	**Clustering method**	**No. of clusters**
Silhouette	0.6325	Manhattan	Hierarchical – Single linkage	2
Baker & Hubert	1	Manhattan	Hierarchical – Single linkage	2
Hubert & Levine	0.0615	Generalized Distance Measure	Hierarchical – Complete linkage	50
		Generalized Distance Measure	Hierarchical - Complete linkage	14
		Generalized Distance Measure	Hierarchical - Complete linkage	12

The optimal distance measure and clustering method using three separate indices are shown along with the associated index value in each case. Where no index metric or value is given, an attempt was made to create more informative clusters rather than optimize a clustering index.

**Figure 5:**
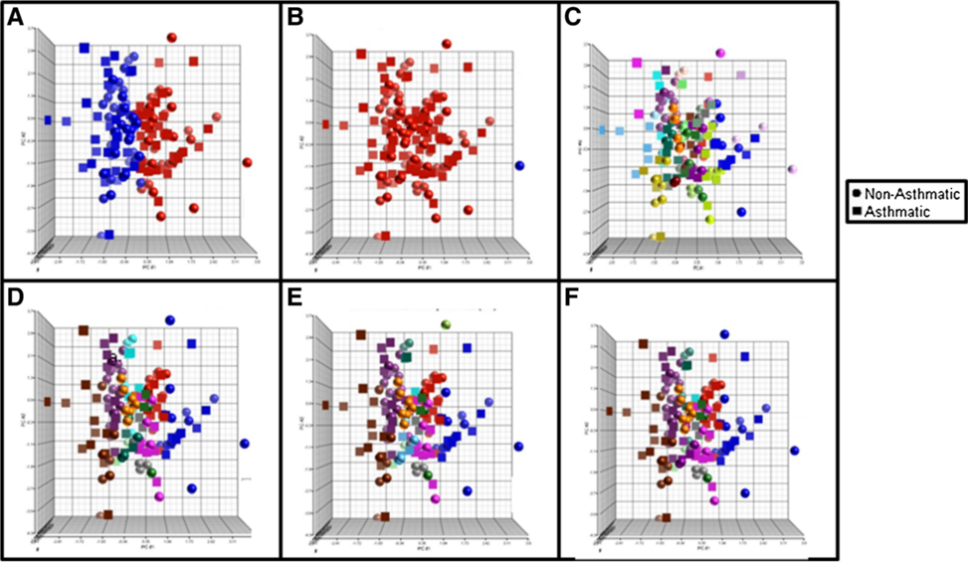
**Scatterplots for each of the 81 Clinical Covariate Clustering Methods (Table**3**); A: Silhouette, B: Baker & Hubert, C: Hubert & Levine, D: Prespecified cluster count = 11, E: Prespecified cluster count = 12, F: Prespecified cluster count = 14.** Colors are representative of individual clusters.

**Figure 6:**
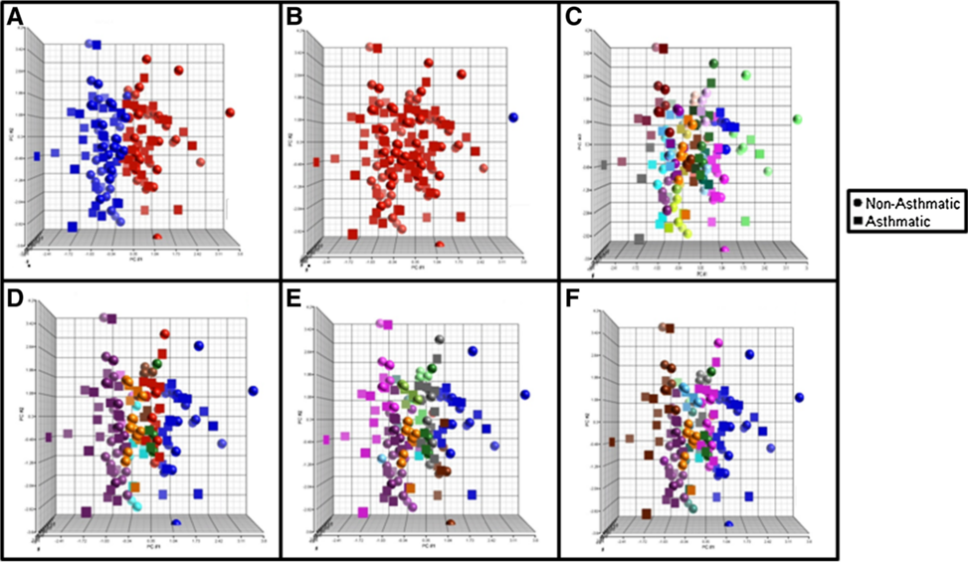
**Scatterplots for each of the 67 Clinical Covariate Clustering Methods (Table**4**); A: Silhouette, B: Baker & Hubert, C: Hubert & Levine, D: Prespecified cluster count = 11, E: Prespecified cluster count = 12, F: Prespecified cluster count = 14.** Colors are representative of individual clusters.

Clinical covariate domain clustering was performed using all 81 clinical covariates and separately with a subset of 67 covariates for comparison to the multi-domain Modk method (see Methods). Cluster validity testing when using 81 covariates suggested that the best clinical chemistry clustering occurred with 2 or 25 (Table 5). The Silhouette clustering yielded two distinct and balanced clusters, however, they were both equally composed of asthmatics and non-asthmatics (Figure 5A). The Baker & Hubert clustering yielded two clusters with one containing a single subject, also seen in the gene expression results (Figure 5B). The Hubert & Levine clustering yielded 25 clusters (Figure 5C). Some of the clusters were all asthmatics and non-asthmatics, however, the well-segregated groups were composed of less than five subjects each (See Additional file 1: Table S2). While this represents a better segregation of asthmatics and non-asthmatics than seen with the gene expression, the group sizes are inadequate for meaningful interpretation. Less optimal clustering combinations were chosen using 81 covariates based on index values to ascertain whether there were cluster counts that were more suitable for asthma segregation and mechanistic interpretation (Figure 5 D-F). These clustering combinations yielded one clearly non-asthmatic cluster and several other equally non-asthmatic and asthmatic clusters. The use of 67 covariates yielded similar results (Table 6, Figure 6, and See Additional file 1: Table S3). One individual was a constant outlier (based on the clustering results) through each of the clustering methods (i.e. the only individual in the cluster). However, this individual did not meet any of the standard metrics for defining outliers. To determine if this potential outlier was deterministic in the clustering results, the individual was removed and the clustering repeated. The resulting clusters still did not segregate asthmatics from non-asthmatics. Since the individual could not be defined as an outlier by any formal test, the original results including this individual are shown.

**Table 5:** Top ranking 81 covariate clustering methods from clusterSim

**Index metric**	**Index value**	**Distance measure**	**Clustering method**	**No. of clusters**
Silhouette	0.6662	Generalized Distance Measure	Partitioning Around Medoids	2
Baker & Hubert	0.9954	Chebyschev	Hierarchical - Single linkage	2
Hubert & Levine	0.0290	Generalized Distance Measure	Hierarchical - Complete linkage	25
		Generalized Distance Measure	Hierarchical - Average linkage	11
		Generalized Distance Measure	Hierarchical - Average linkage	14
		Generalized Distance Measure	Hierarchical - Average linkage	12

The optimal distance measure and clustering method using three separate indices are shown along with the associated index value in each case. Where no index metric or value is given, an attempt was made to create more informative clusters rather than optimize a clustering index.

**Table 6:** Top ranking 67 covariate clustering methods from clusterSim

**Index metric**	**Index value**	**Distance measure**	**Clustering method**	**No. of clusters**
Silhouette	0.6692	Generalized Distance Measure	Partitioning Around Medoids	2
Baker & Hubert	0.9122	Chebyschev	Hierarchical - Single linkage	2
Hubert & Levine	0.0279	Generalized Distance Measure	Partitioning Around Medoids	24
		Generalized Distance Measure	Hierarchical - Average linkage	8
		Generalized Distance Measure	Hierarchical - Average linkage	14
		Generalized Distance Measure	Hierarchical - Average linkage	13

The optimal distance measure and clustering method using three separate indices are shown along with the associated index value in each case. Where no index metric or value is given, an attempt was made to create more informative clusters rather than optimize a clustering index.

Single domain clustering is limited for discovery-focused applications because it only incorporates one domain of data during the analysis stage and relies upon the indicators of health outcome only in the interpretation stage. This requires careful selection of the appropriate variables ahead of time, which reduces the discovery potential of the technique. Both the gene expression and clinical domain clustering are less successful than other methods because of the nature of complex disease with its multiple contributing factors. Even when clustering combinations yielded reasonable clustering (more than two clusters) the clusters had little separation and multiple contributing factors, i.e. large numbers of genes and clinical covariates that defined each cluster. Hence, these unsupervised methods have limited ability to explain the underlying etiology of unknown endotypes.

While clustering approaches were not optimal for the experimental design from this case study, there are examples of the successful use of this approach for defining asthma endotypes. Recent examples relied on *a priori* selection of a limited set of variables with a known relationship to the outcome [22,28,29]. This variable selection method limits the discovery capability of the method, however, and the methods described below try to strike a more even balance between guiding the results via the use of covariates of general relevance without restricting the variables to the point of no longer allowing the data to drive the endotype assignments. Clustering will also be more effective in cases where the molecular differences among the samples collected are heavily influenced by the outcome of interest. For asthma, this would include BALF cells [33] or induced sputum [36]. Those studies also restricted their population to asthmatics thereby increasing the likelihood that changes observed would reflect differences among endotypes but reducing their ability to evaluate the segregation based on separation of asthmatics and non-asthmatics. While asthma does provide an opportunity for evaluating samples collected directly from the target organ in human subjects, many complex diseases do not, so methods that can leverage blood and other surrogate matrices are important.

### Multiple domains

Given that existing methods are suboptimal when attempting to define novel disease endotypes, we developed a multi-step decision tree method that attempts to integrate clinical markers, gene expression data, and disease indicators for endotype discovery. The method was designed to maximize the links between the resulting endotypes and the clinical markers and genes most associated with that endotype. For comparison, we also tried the Modk-prototypes algorithm, which has previously been used successfully to link gene expression and clinical markers to histopathology results in response to chemicals.

The decision tree method incorporates gene expression and clinical chemistry in a preprocessing stage, disease status and gene expression in an analysis stage, and all three data domains in the interpretation stage (Figure 2). This method seeks to balance the influence of clinical covariates and disease status to maximize the probably of identifying disease mechanisms while simultaneously minimizing the bias against novel biological processes. The incorporation of covariates with known association to disease in the preprocessing step guides the clustering of genes to disease-relevant pathways without explicitly including the disease status. The disease status is incorporated in the analysis stage to identify those gene clusters that best segregate asthmatics into distinct groups.

To enhance our ability to interpret the mechanistic basis underlying different asthma endotypes, the information contained in each cluster of genes was first summarized using Principal Components Analysis (PCA) prior to our considering the asthma phenotype (Figure 3). Starting with the clusters as crude groupings of associated genes, we used PCA to segregate each of these into several principal components (PCs), thus resolving the clusters into subclusters. Another option would have been to set a lower cut point on the original dendrogram; however, we chose not to use that approach because a cut point that low on the tree would likely separate many genes that should be considered together. The PCA method, however, provided a “fuzzy” sub-clustering within our main clusters, that is, each gene had the opportunity to contribute to more than one PC.

The Modk-prototypes algorithm partitions subjects into their respective disease class groups using k-means and k-modes clustering over three subjectively weighted data domains : gene expression, clinical chemistry, and histopathology or disease status [12,44]. For this study, the three domains of data were blood gene expression data, the clinical covariates, and the allergy and asthma health outcome indicators. This represents the only completely integrated analysis that incorporates all three data streams in a single step (Figure 2). As with the decision tree method, genes and clinical covariates that heavily influence the clusters are readily obtained, though the direction and magnitude of the influence is less readily extracted. The influence of disease status can be adjusted when using a pre-defined weighting scheme, but this introduces the problem of how to choose a weighting scheme *a priori*. For this study, the Modk-protypes method was performed using seven pre-defined summative weighting schemes and one weighting scheme determined via adaptive weighting based on the maximization of the objective function (Table 3).

The classification accuracy of the various weighting schemes is compared with that for the decision tree method in Table 7. Four Modk-prototypes weighting schemes yielded 65% or better accuracy, but the overall accuracy of the decision tree was the highest at 78%. Unlike the single-domain clustering methods, both the Modk-prototype weighting schemes and decision tree optimally yielded between 7 and 12 clusters with approximately half asthmatic clusters in all cases (Table 7). The accuracy of each asthmatic cluster or leaf from those methods resulting in at least 65% overall accuracy are shown in Table 8. Low accuracy may be indicative of a cluster that could be split into separate groups or a cluster for which the data are insufficient to describe the mechanistic basis of disease in those individuals. While the decision tree had a better overall accuracy, the performance of a couple of the Modk-prototypes weighting schemes was slightly better when considering only the more highly accurate asthmatic clusters. While this is arguably more important than overall accuracy since the goal is mechanistic insight rather than development of a classifier, how to objectively select the optimal weighting scheme is a persistent challenge.

**Table 7:** Accuracy per Method/Weighting scheme

**Method**	**Classification %**	**Groups Asthmatic/Non-Asthmatic**
**33/33/33**	**65**	**4/5**
**20/40/40**	**66**	**3/4**
**40/20/40**	**67**	**6/6**
50/50/0	56	2/4
30/60/10	61	3/3
60/30/10	63	4/8
**40/40/20**	**66**	**4/8**
Adaptive	60	1/3
**Decision**	**78**	**4/4**

The weighting schemes are shown as Gene Expression Domain Weighting/Clinical Covariate Domain Weighting/Indicators of Disease Status Domain Weighting. Column 2 shows the percentage of subjects correctly classified by asthma status after assigning asthma status based on the majority of subjects in the group. Column 3 shows the number of asthmatic and non-asthmatics groups respectively using this definition. Entries in bold represent those methods showing at least 65% accuracy. Information on the classification accuracy of the individual asthmatic clusters for these methods is shown in Table 8.

**Table 8:** Accuracy per asthmatic leaf for each Modk weighting scheme and the multi-step decision tree method

**Weighting scheme**	**Cluster/Leaf number**	**% Correct**
33/33/33	1	84
	3	58
	5	88
	8	67
20/40/40	2	68
	3	83
	4	67
40/20/40	1	73
	2	90
	4	55
	5	100
	6	56
	8	57
40/40/20	3	86
	5	70
	6	60
	12	100
Decision Tree	1	90
	2	71
	5	60
	8	73

The weighting schemes are shown as Gene Expression Domain Weighting/Clinical Covariate Domain Weighting/Indicators of Disease Status Domain Weighting. Methods with at least 65% overall accuracy were evaluated based on the accuracy of the individual asthma groups. Column 2 shows the cluster number from the original output. Missing numbers represent non-asthmatic clusters. Column 3 shows the percentage of asthmatics in each asthma group.

The multiple domain methods were better able to segregate asthmatics and non-asthmatics compared to the single-domain methods because disease status information was explicitly used to define the subject groups. The importance of incorporating health outcome indicators becomes apparent by comparing the accuracy of the resulting cluster from the seven differing weighting schemes. The weighting schemes that incorporate health outcome indicators at less than 20% are less successful than those that incorporate them at higher percentages. In contrast, even when the percentage of weight given to gene expression and clinical covariates are reversed, there is no significant gain in accuracy with regard to the percentage of asthmatics that are correctly labeled as asthmatic by the Modk-prototypes method.

In comparison to the other methods, multiple domain methods provided the greatest information with respect to identifying asthma endotypes. The two multiple domain methods differ in how and at what stage the domains of data are incorporated (Figure 2). The two methods give similar results, groups of asthmatics and non-asthmatics based on the indicators of health outcome. Each method distributes the subjects slightly differently across different leaves and clusters (See Additional file 1: Table S4A-J). However, the decision tree and Modk-prototypes methods agree 84% of the time with regard to asthmatic or non-asthmatic asthma calls.

Modk-prototypes are able to classify both asthmatic and non-asthmatics with some success. However, understanding the variables that contribute to each cluster is as difficult as with single-domain clustering. The resulting prototypes can be analyzed using methods such as factor or principal components analysis; however, there are many variables that contribute to each cluster. Each weighting scheme resulted in a defined list of key genes that contributed to the clustering. This list of key genes was not duplicative between the higher accuracy weighting schemes leading to additional difficulty in the interpretation process. If one weighting scheme was considerably more accurate or if the adaptive weighting scheme had been as accurate, it would be possible to use the key gene list to reduce the numbers of genes considered in the interpretation stage or focus the interpretation. To select a single user-defined weighting scheme under these circumstances, however, could have introduced a user bias in the interpretation step.

The Modk method was originally designed to cluster biological samples (i.e., microarray gene expression data, phenotypic variables such as clinical chemistry evaluations, and histopathology observations) from perturbation studies in target organs or studies of disease where there is an acute event, i.e., chest pain for the study of heart disease. The MICA study, however, was an observational study where no acute event occurred before sample collection. Gene expression in blood cells reflects many influences (e.g., time and content of your last meal, medications), most of which have nothing to do with disease [49]; of disease related influences, only a subset will relate specifically to asthma. Due to the additional variability in MICA attributed to the use of blood gene expression in lieu of lung gene expression and lack of an acute event before data collection, the decision tree method outperformed the Modk method in this study. However, the Modk method would likely outperform the decision tree method in cases better matched to its intended experimental design.

The multi-step decision tree method has several advantages for endotype discovery and characterization for experimental designs like the MICA childhood asthma study. This method showed the best segregation of asthmatics from non-asthmatics of all methods tested. Moreover, the decision tree method is advantageous because of its incorporation of clinical and gene expression data into the preprocessing stage, which leads to the reduction of the dataset to 380 gene variables. This dimension reduction gives the method more power while retaining the structure of the gene expression data in relation to the clinical covariate data. Further, the multi-step decision tree method retains the information used to determine each of the leaves. This characteristic makes them much easier to interpret than less transparent methods. By aggregating the gene expression and clinical data prior to generating the decision tree, we enhance this interpretability by providing a rich set of biological information from which to define potential mechanisms underlying the segregation of endotypes. Multiple domain methods, multi-step decision tree, and Modk, are all somewhat reliant on the varied manner in which asthma status was defined in this case study, but they have the flexibility to overcome labeling errors that cause too much noise in other methods, e.g., t-test method. Subsequent analysis has also shown that the decision tree method can distinguish known endotypes of childhood asthma in a purely data-driven manner while highlighting potentially novel endotypes (Reif et al., in preparation).

The multi-step decision tree approach was superior to all other methods for the case study considered here. However, this method should be cautiously applied to other complex disease problems. For example, the multi-step decision tree method can be severely limited by incorrectly selecting the clinical covariates for the analysis. The exclusion of important or inclusion of misleading covariates may bias the selected gene expression data. Each clinical covariate must be considered individually for accuracy and probable relationship to disease processes. Highly correlated markers can also unduly influence the clustering of the gene expression data and bias the resulting metagenes in favor of certain biological processes. Conversely, reducing the clinical covariates based solely on correlation can ignore distinct biological processes where the correlation is informative for endotype definition. The key is to ensure that the clinical measures are not biased towards selecting too many covariates related to one disease process, e.g., selecting only clinical markers of inflammation. Further, it is important not to have duplicative measures of one clinical marker, such as measures of the same metabolite in urine and blood, unless there are duplicative measures for all clinical markers because the gene expression data could, as a result, be biased. We used a range of clinical variables including immunological, blood chemistry and hematological indicators and attempted to reduce correlated markers without restricting the biological space being interrogated.

### Implications for GWAS, EWAS, and exposomics

In the postgenomic era, the primary focus of complex disease-related research such as age-related macular degeneration (AMD), Type I and II diabetes, inflammatory bowel disease, cardiovascular disease, and asthma has been on understanding the genetic susceptibility and heritability of the disease. Genome-wide association (GWAS) mapping and other similar efforts have been applied with increasing success [50-54]. Despite these rapid advances, challenges remain in interpreting the massive amount of genomic variation data to 1) identify susceptibility genes associated with particular complex diseases and 2) understand the underlying etiology of the complex diseases. Beyond GWAS approaches, limited insight has been gained by applying other genetic approaches to study complex disease [55]. There are innate limitations to studies that consider only genetic data, mainly because contributions from single genes are often limited and genetic studies generally do not offer clues about the functional context of a gene associated with a complex disorder. Approaches like the multi-step decision tree described here should complement the ever-growing number of genetic associations by providing additional mechanistic information for interpreting those results. In addition, a better definition of mechanistically distinct subtypes of complex diseases will greatly enhance the power and value of the GWAS approach [56].

The search for causal factors in complex diseases is also complicated by the fact that genetic drivers likely account for less than 30% of the increased risk for those individuals who develop common diseases in western society [57]. Wild has proposed the concept of an exposome that represents all environmental (i.e. non-genetic) contributors to disease received by an individual during life [58]. Enthusiasm for this concept has increased in large part due to emerging technologies that provide this new generation of exposure information [59]. Rappaport and Smith further consider the exposome paradigm as a comprehensive and quantitative view of environmental exposure to include biomarkers representing internal processes such as inflammation, lipid peroxidation, and preexisting disease [60]. They propose a strategy that would measure all chemicals (or products of downstream processing or effects, i.e., read-outs or signatures) in a subject’s blood. Although few studies have applied a true discovery-based exposomic approach [61], methods are being investigated [62,63] and momentum is building around this concept [64]. As EWAS [62] and exposome [58], studies become the norm, they will complement GWAS studies and provide a more complete picture of underlying drivers of disease.

As experiments are designed to integrate these data streams, several aspects from the current case study should be considered. Measurements chosen for relevance to the disease being studied are needed in addition to top-down driven strategies for novel biomarker discovery. These measurements will help to ground the analysis as they did for the gene expression data in this study. In this study, clinical covariates with known or suspected relationships to asthma were used to improve the analysis. By leveraging covariates that are expected to relate in some way to asthma, we are able to identify those gene expression changes that are relevant for our phenotype without sacrificing the exploratory nature of the study. Defining endotypes for any complex disease will be an iterative process by which additional informative covariates are added to each study as we better understand the disease. The incorporation of metabolomic and proteomic measures, as proposed for the exposome, will increase the discovery potential and minimize the bias introduced by the directed covariate selection. The most important aspect, however, is the data-driven nature of the disease characterization. Discovery-driven methods such as the multi-step decision tree, will make it possible to move towards more mechanism-based clinical biomarkers of disease and thereby reduce our reliance on diagnostic criteria that are tied too closely to symptoms. In fact, it has been said for asthma that the biggest advance yet to come is not the realization of “personalized medicine” but instead the “better characterisation of the phenotype that can be linked to genetic characterisation” [65].

### Compatibilities with other systems biology approaches

For this case study, we considered simple correlations rather than more advanced statistical assessments in determining gene/covariate associations [56,66,67]. Given the other factors being studied, the selection of a simple correlation metric allowed us to focus on the unique aspects of this approach without the additional considerations of more sophisticated association methods. Bayesian networks [68-70], Boolean networks [71-73], and mutual information criteria [69,74-76] have all been successfully used in similar situations, and the analysis methods described here should be compatible with all of these methods. Bayesian approaches in particular are attractive in their ability to incorporate prior information [77,78] as has been demonstrated with genetics studies [79]. As we consider the iterative nature of this analysis, the incorporation of prior information in a Bayesian framework seems a logical next step.

## Conclusions

A better understanding of complex disease relies on the integration of all available domains of data. There are gene-gene, gene-covariate, covariate-covariate interactions that are lost when one variable is considered at a time (t-test) or one domain at a time (single-domain clustering). The decision tree method applies these interactions between the domains as a tool for dimension reduction resulting in a more accurate model that is easily interpretable. The application and usefulness of models created to increase understanding of complex disease are dependent on the available data and the manner in which these data are incorporated into the model (Figure 2). In particular, much care was taken to incorporate both the covariate information and asthma status in a way that would guide discoveries toward those most informative for distinguishing asthma endotypes while at the same time minimizing the bias against discovery of novel mechanisms associated with a heavy reliance on known asthma etiology.

Several considerations drove the development of the multi-step decision tree method. The first consideration was the segregation of known asthmatics and non-asthmatics. The earlier in the analysis the covariate on which you want to segregate is introduced, the more influence it will have and the more likely that optimal segregation will be achieved. However, this must be balanced with avoiding undue influence on the natural segregation of individuals based on the mechanistic drivers of interest. We adopted a multi-step analysis pipeline largely to address this particular issue. The second consideration was ease of interpretation. Decision trees are particularly powerful with respect to interpretation since the process is completely transparent to the user. The final consideration was the amount of mechanistic information revealed by the different methods. By pre-assembling our gene expression and covariate information, we carried all gene annotation and knowledge regarding the biological pathways associated with the covariates into the interpretation phase. This greatly increased our ability to translate the branches from our decision tree into biological insights. As a result, we believe our multi-step decision tree method represents a novel approach to the discovery of new mechanisms underlying complex disease and complements existing methods that convert these mechanistic insights into informative biomarkers for the diagnosis of disease.

## Availability of supporting data

The microarray data from this publication have been submitted to the Gene Expression Omnibus (GEO) repository (http://www.ncbi.nlm.nih.gov/geo/) and assigned the identifier GSE35571.

## Abbreviations

MICA: Mechanistic indicators of childhood asthma; PCA: Principal components analysis.

## Competing interests

The authors declare no competing interests.

## Authors’ contributions

CRW, SWE, and DMR designed the experiments; JEG, ECH, and EEH designed the MICA study. CRW, DMR, and PRB completed the analysis. CRW and SWE interpreted the results. All authors contributed to editing this manuscript. All authors read and approved the final manuscript.

## Supplementary Material

Additional file 1 Supplemental Tables and Figure; File containing supplemental tables and figures referenced in the text. Click here for file
