# Supplementary material for: Decision tree-based method for integrating gene expression, demographic, and clinical data to determine disease endotypes
Source: BMC Syst Biol. 2013 Nov 4;7:119. doi: 10.1186/1752-0509-7-119 (PMC4228284; doi:10.1186/1752-0509-7-119)
Supplement: Additional file 1 — Supplemental Tables and Figure; File containing supplemental tables and figures referenced in the text. [file 1752-0509-7-119-S1.pdf]

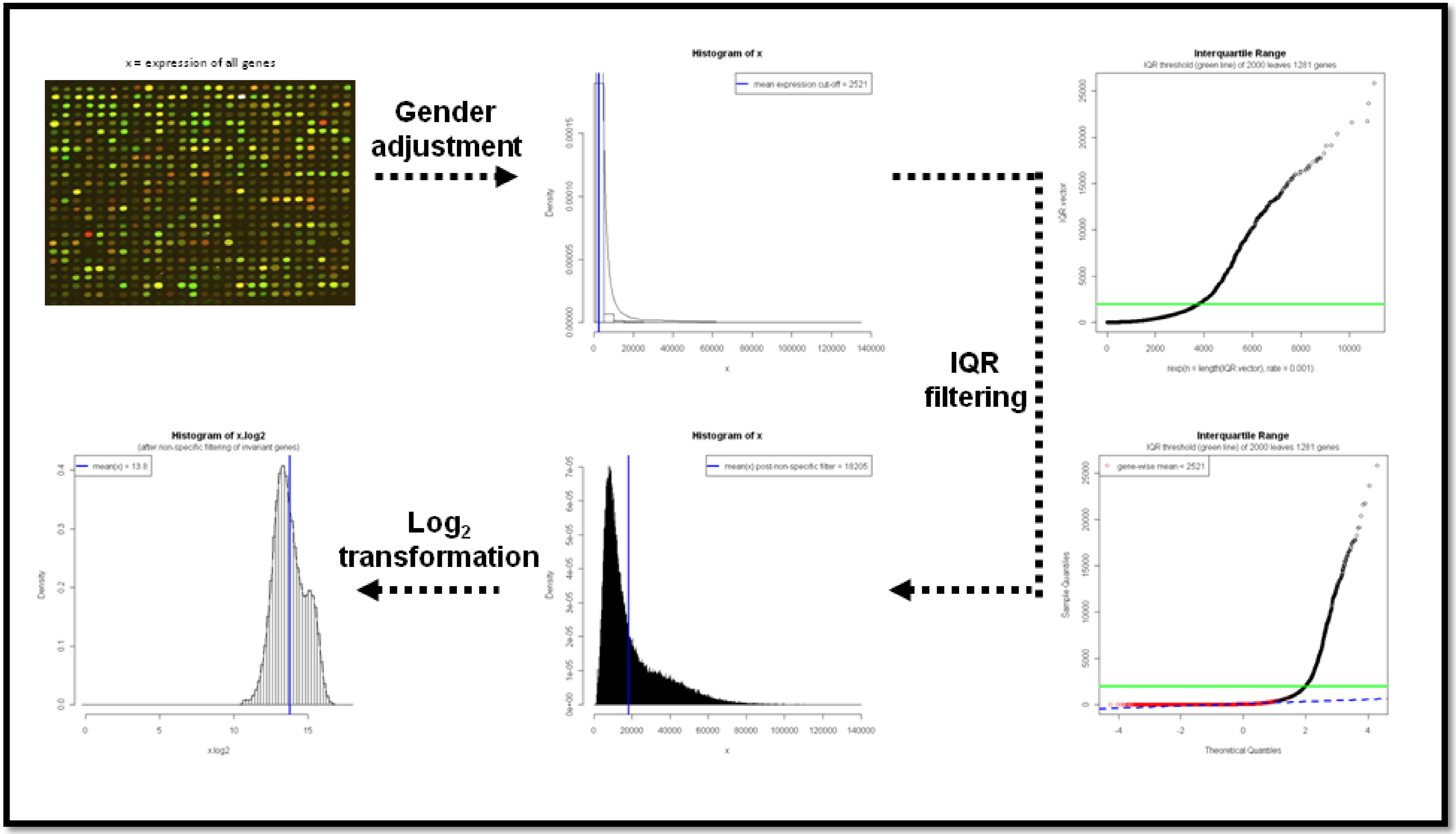

Supplemental Figure 1. Preprocessing of Gene Expression Data and Gene Selection

Supplemental Table 1 Quantiles for Allergens converted to Indicator of Disease Status

Level of Allergy	Phadiatop	Fx5FoodScreen
Not detectable	<0.39	<0.35
Mildly Allergic	(.390,4.75]	[0.36-0.60]
Allergic	(4.745, 22.00]	(0.60,1.23]
Highly Allergic	(22.000, 50.40]	(1.23,2.81]
Severely Allergic	(50.400, 101.000]	(2.81,101.0]

Brackets indicate the precise range for each category: (not inclusive, [inclusive.

Supplemental Table 2. Number of Asthmatics and Non-Asthmatics in each cluster (81 Covariate List)

Clustering Method	Number in Cluster	Non-Asthma/Asthma
Silhouette	69	32/37
	77	42/35
Baker & Hubert	145	73/72
	1	1/0
Hubert & Levine	7	2/5
	14	7/7
	4	3/1
	12	9/3
	5	4/1
	6	2/4
	5	0/5
	2	0/2
	2	1/1
	5	3/2
	11	4/7
	9	5/4
	6	0/6
	3	3/0
	3	½
	3	2/1
	13	4/9
	7	3/4
	5	4/1
	1	1/0
	4	3/1
	11	6/5
	2	1/1
	4	4/0
	2	2/0
Less Optimal Method 1	22	8/14
	21	12/9
	4	2/2
	28	15/13
	9	8/1
	12	6/6
	20	7/13
	13	6/7
	7	5/2
	3	1/2

	7	4/3
Less Optimal Method 2	22	8/14
	20	11/9
	4	2/2
	28	15/13
	9	8/1
	1	0/1
	20	7/13
	13	6/7
	7	5/2
	3	½
	4	2/2
	7	4/3
	7	4/3
	1	1/0
Less Optimal Method 3	22	8/14
	21	12/9
	4	2/2
	28	15/13
	9	8/1
	1	0/1
	20	7/13
	13	6/7
	7	5/2
	3	½
	11	6/5
	7	4/3

Number of asthmatics and non-asthmatics in each cluster using each clustering combination from clusterSim organized by validity index measure for the 81 Covariate List.

Supplemental Table 3 Number of Asthmatics and Non-Asthmatics (67 Covariate List)

Clustering Method	Number in Cluster	Non-Asthma/Asthma
Silhouette	69	32/37
	77	42/35
Baker & Hubert	145	73/72
	1	1/0
Hubert & Levine	4	2/2
	10	4/6
	3	½
	6	5/1
	7	5/2
	9	4/5
	8	4/4
	13	5/9
	4	0/4
	9	6/3
	3	½
	8	6/2
	6	1/5
	1	1/0

	4	1/3
	4	4/0
	8	5/3
	4	1/3
	6	1/5
	6	3/3
	4	3/1
	6	3/3
	6	3/3
	7	5/2
	23	10/13
Less Optimal Method 1	32	15/17
	5	3/2
	48	22/26
	17	12/5
	11	6/5
	7	5/2
	3	1/2
	3	2/1
Less Optimal Method 2	24	11/13
	5	3/2
	28	15/13
	10	8/2
	1	0/1
	8	4/4
	19	6/13
	20	8/12
	7	5/2
	3	1/2
	10	6/4
	1	1/0
	7	4/3
	3	2/1
Less Optimal Method 3	32	15/17
	5	3/2
	28	15/13
	10	8/2
	1	0/1
	19	6/13
	20	8/12
	7	5/2
	3	1/2
	10	6/4
	1	1/0
	7	4/3

Number of asthmatics and non-asthmatics in each cluster using each clustering combination from clusterSim organized by validity index measure for the 67 Covariate List.

Supplemental Table 4a. Overlap of cluster membership between two MODK weighting schemes

		33/33/33								
		1	2	3	4	5	6	7	8	9
20/40/40	1	0	7	0	0	0	0	0	2	5
	2	0	2	0	5	9	0	2	1	6
	3	10	0	2	0	0	0	0	0	0
	4	5	2	8	0	0	3	0	0	0
	5	0	0	2	0	0	0	15	0	0
	6	0	11	0	0	0	17	0	0	1
	7	4	0	0	14	0	1	4	0	8

Supplemental Table 4b. Overlap of cluster membership between two MODK weighting schemes

		40/40/20											
		1	2	3	4	5	6	7	8	9	10	11	12
20/40/40	1	0	4	0	0	1	0	4	1	4	0	0	0
	2	3	0	11	1	0	0	2	0	5	0	3	0
	3	0	0	2	0	0	0	3	0	0	1	0	6
	4	0	0	0	0	7	4	4	0	0	2	0	1
	5	0	0	0	0	0	1	4	0	0	0	12	0
	6	0	0	0	0	2	0	6	20	1	0	0	0
	7	2	0	1	0	0	0	0	0	12	11	4	1

Supplemental Table 4c. Overlap of cluster membership between two MODK weighting schemes

		40/20/40											
		1	2	3	4	5	6	7	8	9	10	11	12
20/40/40	1	1	0	2	1	1	0	3	4	1	0	1	0
	2	0	7	0	1	0	0	0	6	6	1	4	0
	3	5	3	3	1	0	0	0	0	0	0	0	0
	4	1	0	3	3	1	5	3	0	0	0	0	2
	5	0	0	2	1	0	0	0	0	0	0	14	0
	6	3	0	0	4	3	4	7	0	0	0	0	8
	7	5	0	0	0	0	0	0	4	10	8	1	3

Supplemental Table 4d. Overlap of cluster membership between two MODK weighting schemes

		33/33/33								
		1	2	3	4	5	6	7	8	9
40/40/20	1	0	0	0	5	0	0	0	0	0
	2	0	1	0	0	0	0	0	1	2
	3	2	0	0	3	7	0	1	0	1
	4	0	0	0	0	1	0	0	0	0
	5	2	2	2	0	0	4	0	0	0
	6	0	0	5	0	0	0	0	0	0
	7	1	14	5	0	0	0	3	0	0
	8	0	5	0	0	0	16	0	0	0
	9	0	0	0	2	0	0	2	1	17
	10	6	0	0	7	0	1	0	0	0
	11	0	0	0	2	1	0	15	1	0
	12	8	0	0	0	0	0	0	0	0

Supplemental Table 4e. Overlap of cluster membership between two MODK weighting schemes

		33/33/33								
		1	2	3	4	5	6	7	8	9
40/20/40	1	9	0	0	2	0	2	0	0	2
	2	3	0	0	1	6	0	0	0	0
	3	2	1	5	0	0	0	1	0	1
	4	0	6	5	0	0	0	0	0	0
	5	1	2	0	0	0	2	0	0	0
	6	2	1	1	0	0	5	0	0	0
	7	0	10	1	0	0	2	0	0	0
	8	0	1	0	0	0	0	1	2	10
	9	0	0	0	13	2	0	1	0	1
	10	0	0	0	2	0	0	1	0	6
	11	0	1	0	0	1	0	17	1	0
	12	2	0	0	1	0	10	0	0	0

Supplemental Table 4f. Overlap of cluster membership between two MODK weighting schemes

		40/20/40											
		1	2	3	4	5	6	7	8	9	10	11	12
40/40/20	1	0	0	0	0	0	0	0	0	5	0	0	0
	2	0	0	2	0	0	0	0	1	1	0	0	0
	3	0	9	0	0	0	0	0	1	3	0	1	0
	4	0	0	0	0	0	0	0	0	1	0	0	0
	5	0	0	2	0	4	3	0	0	0	0	0	1
	6	0	0	0	4	0	0	1	0	0	0	0	0
	7	1	0	4	7	0	1	6	1	0	0	3	0
	8	2	0	0	0	1	4	6	0	0	0	0	8
	9	3	0	0	0	0	0	0	11	0	8	0	0
	10	3	1	0	0	0	1	0	0	4	1	0	4
	11	0	0	0	0	0	0	0	0	3	0	16	0
	12	6	0	2	0	0	0	0	0	0	0	0	0

Supplemental Table 4g. Overlap of MODK cluster membership with leafs of decision tree

		20/40/40						
		1	2	3	4	5	6	7
Decision Tree	1	2	5	7	2	0	6	8
	2	1	6	2	4	0	1	0
	3	1	0	0	1	6	3	4
	4	0	0	0	3	0	5	6
	5	1	0	0	4	0	9	1
	6	6	5	1	0	4	4	9
	7	1	4	2	1	3	0	3
	8	2	5	0	3	4	1	0

Supplemental Table 4h. Overlap of MODK cluster membership with leafs of decision tree

		33/33/33								
		1	2	3	4	5	6	7	8	9
Decision Tree	1	9	1	1	4	1	5	1	2	6
	2	1	3	4	0	3	0	0	0	3
	3	0	3	2	1	0	0	6	1	2
	4	2	1	0	3	0	8	0	0	0
	5	3	4	2	0	0	6	0	0	0
	6	1	7	0	7	2	1	5	0	6
	7	2	1	1	4	0	0	4	0	2
	8	1	2	2	0	3	1	5	0	1

Supplemental Table 4i. Overlap of MODK cluster membership with leafs of decision tree

		40/40/20											
		1	2	3	4	5	6	7	8	9	10	11	12
Decision Tree	1	1	0	4	0	3	0	2	4	8	4	1	3
	2	0	1	4	0	1	2	4	0	1	0	0	1
	3	0	1	0	0	1	2	3	0	3	0	5	0
	4	0	0	0	0	3	0	0	5	1	5	0	0
	5	0	0	0	0	1	0	3	8	0	1	0	2
	6	0	1	3	1	0	0	5	3	7	4	5	0
	7	4	0	0	0	0	1	1	0	2	0	4	2
	8	0	1	3	0	1	0	5	1	0	0	4	0

Supplemental Table 4j. Overlap of MODK cluster membership with leafs of decision tree

		40/20/40											
		1	2	3	4	5	6	7	8	9	10	11	12
Decision Tree	1	6	3	1	1	4	0	0	5	4	2	1	3
	2	0	3	3	3	0	0	1	2	1	1	0	0
	3	0	0	0	3	1	0	1	3	1	1	5	0
	4	4	0	0	0	0	2	1	0	0	0	0	7
	5	2	0	2	1	0	3	4	0	0	0	0	3
	6	1	2	1	2	0	1	4	2	6	5	5	0
	7	2	0	0	1	0	0	1	2	5	0	3	0
	8	0	2	3	0	0	3	1	0	0	0	6	0
